# Environmental life cycle assessment of rice production in northern Italy: a case study from Vercelli

**DOI:** 10.1007/s11367-022-02109-x

**Published:** 2022-10-26

**Authors:** Vinci Giuliana, Maddaloni Lucia, Ruggeri Marco, Vieri Simone

**Affiliations:** Department of Management, University of Rome, Via del Castro Laurenziano 9, 00161 Sapienza, Italy

**Keywords:** Rice, Agricultural emissions, Crop management, Multivariate analysis, PCA, Piedmont

## Abstract

**Purpose:**

The study’s objective is to assess the environmental performance of rice production in Northern Italy, in particular in Piedmont, the first Italian and European district for the rice-growing area, and thus identify the most critical hotspots and agricultural processes. In particular, as a case study, a farm located in Vercelli (VC) has been chosen. Subsequently, the study results were compared with other different cultivation practices to evaluate the most sustainable choice.

**Methods:**

The application of the LCA has been performed, highlighting the phases of rice production that have the most significant impact. Then, uncertainty and sensitivity analyses have been made to estimate the robustness of the results and assess the influence of changing some input variables on emission reduction. Finally, multivariate statistical, specifically a principal component analysis (PCA), was conducted to aid the interpretation of the output dataset of this case study. LCA, uncertainty analysis, and sensitivity analysis were performed with SimaPro 9.2.0, using ReCiPe 2016 Midpoint (H) methodology, and PCA with R software.

**Results and discussions:**

The hotspot with the highest environmental load is irrigation, which compared to the other phases impacts more in 15 out of 18 categories, including 12 with impacts greater than + 75%. This is because irrigation causes direct impacts, related to the methanogenesis in rice fields, but also indirect impacts related mainly to the production of the energy mix required to move the large masses of irrigation water. Therefore, different water management systems were compared and results show that the irrigation systems based on intermittent paddy submergence (DSI) could result in − 40% lower impacts, resulting to be the preferable technique over the other irrigation systems analyzed, including the traditional one used in this study.

**Conclusions:**

In order to reduce the environmental impacts related to the irrigation process, a water management system characterized by intermittent flooding of the paddy field (DSI) could be used as it reduces the environmental impacts the most (− 40%), while the least suitable system is one characterized by continuous flooding without drought periods, as it causes the highest impacts.

**Supplementary Information:**

The online version contains supplementary material available at 10.1007/s11367-022-02109-x.

## Introduction

### Background

The agro-food sector is one of the main responsible for determining adverse effects on the environment. Therefore, world governments, driven by the United Nations (UN), are shifting their policies toward more sustainable production. In recent years, to comply with the objectives of the 2030 Agenda, a focus has been placed on the environmental impact of different agricultural productions. Among the agronomic crops, those most studied from the environmental point of view are corn, wheat, and rice as they are the three most grown, produced and consumed globally. Rice (*Oryza sativa L. spp*), which in 2019 accounted for 25% (755 million tons) of world cereal production (2.97 billion tons) (Faostat 2020), is the most widely used for human consumption, with about 50% of the world’s population dependent on this crop. Globally, the largest rice-producing Countries are China (29%), India (24%), Indonesia (7%), and Bangladesh (7%) (Faostat 2020). More than half of world production, therefore, is concentrated in two countries (China and India), and about 95% in developing countries (DCs), that are highly dependent on this cereal, for whom it is a key component of their diets and economies. However, rice is also cultivated in industrialized countries (ICs), especially in Europe, where about 8 million hectares (2020) are dedicated to the cultivation of this cereal, for a production of about 3 million tons. Even if far from the world’s leading producers, Italy is Europe’s largest rice producer. It has a cultivated area of about 500 thousand hectares in 2020 and a production of 1.5 million tons (53% of European production) (Enterisi 2021; Istat 2021), making rice production one of the main Italian agri-food sectors. In particular, Piedmont is the most important rice-growing district, and about 50% of Italy’s rice-growing area is located in the provinces of Vercelli (70,000 hectares), Novara (30,000), Alessandria (8000), and Biella (4000), as well as a few small crops in the provinces of Cuneo and Turin (Istat 2021). In addition, Piedmont produces Italy’s only Protected Designation of Origin (PDO) rice, namely “*Baraggia Biellese and Vercellese*” (Consorzio Baraggia 2021). Therefore, rice represents for this geographical area one of the leading crops in the agri-food sector, and its production plays a positive role both economically and socially as it could create wealth and jobs. However, the agronomic practices adopted in Italy for its cultivation can have disadvantages in terms of environmental impacts (Zoli et al. 2021). In fact, unlike Asian Countries that are rich in water resources, as they receive a very high annual rainfall (Masuda 2019) and sometimes have a dense network of streams and rivers (Harun et al. 2021) causing their water sources to be sufficient to meet the water needs necessary for rice cultivation, Italian soil and climate conditions lead to a type of irrigated cultivation characterized by continuous flooding through water administration. This, therefore, causes additional impacts, related to the production process of electricity used to pump water, in addition to methane (CH_4_) emissions due to the fermentation of organic matter under anaerobic conditions (IPCC 2006; Liu and Whitman 2008). Methane is the second most important greenhouse gas (GHG) after carbon dioxide (CO_2_), and although it has a shorter life (12 years), it traps heat 25 times longer. Since pre-industrial times, it has been responsible for about 20% of global warming, and 7–17% of atmospheric methane is emitted by rice fields, which, with about 25–100 million tons per year, are the largest source of anthropogenic CH_4_ (Kirschke et al. 2013; Su et al. 2015). Moreover, conventional rice production requires the massive application of plant protection products, especially herbicides, negatively affecting the environment, human health, and natural resources (Fusi et al. 2014), due to the emission of nitrous oxide (N_2_O), another GHG with 298 times more climate-changing power than CO_2_. For all these reasons, rice production is among the activities with the highest environmental impacts in the agro-food sector, and the focus on reducing GHGs in rice production is a priority. Therefore, in this context, the identification and assessment of the most impactful activities is the first step toward more sustainable decision-making in the agro-food sector and towards mitigating the carbon footprint of rice production. For investigating the environmental performance of a product, service, system, or activity, the life cycle assessment (LCA) is the most widely used methodology. It is a standardized method referring to ISO 14040:(2006b) and ISO 14042:(2006a) that allows the assessment of the environmental, economic, and social impacts related to a product (process and/or service). Initially, it was developed to analyze industrial systems, but it is also widely applied in other sectors such as the agri-food one (Notarnicola et al. 2012; Payen et al. 2018). This field is mainly used to highlight environmental hotspots, i.e., all those activities or processes responsible for environmental impacts or to compare different agronomic techniques implemented in the same crop.

### State of the art

The application of LCA has been used to evaluate which were the most suitable agro-systems to produce rice concerning the environmental aspect. Most studies on the application of LCA in rice production are mostly concentrated in Asian Countries: Thailand (Jirapornvaree et al. 2021; Yodkhum et al. 2018), China (Xu et al. 2022; Shen et al. 2021), Malaysia (Abdul Rahman et al. 2019; Harun et al. 2021), Iran (Habibi et al. 2019; Morandini et al. 2020), Bangladesh (Shew et al. 2019; Jimmy et al. 2017), and Japan (Masuda 2019). Jirapornvaree et al. (2021), for example, consider complete rice production (from cradle to gate), showing that the total GWP is about 38 kg CO_2_ eq, while Yodkhum et al. (2018) study different waste straw management systems. In China, Xu et al. (2022) evaluate the environmental impacts of a monoculture rice crop with an integrated rice-shrimp crop, while Shen et al. (2021) take Ratoon rice as the object of study so as to identify its hotspots. In both studies, production and field emissions as a result of nitrogen fertilizers are found to be the predominant sources of environmental impacts. In Malaysia, Abdul Rahman et al. (2019) study a conventional crop, establishing how 76% of emissions (out of a total GWP of 1390 kg CO_2_ eq × 1 ton) come from field emissions, while Harun et al. (2021) compare an organic crop with a conventional one, agreeing that the latter is more sustainable precisely because of the absence of fertilizers. In Iran, on the other hand, Morandini et al. (2020), show how reduced application of chemical fertilizers and pesticides, as well as improved rotational management practices, led to less use of human power, machinery, and fuel, resulting in lower total emissions, while Habibi et al. (2019) conduct their study comparing transgenic and non-transgenic rice genotypes, however, without specifying the boundaries of the study. Remarkable, however, is the study by Shew et al. (2019), who analyze two rice crops in three different seasons, with three levels of precipitation. The results show that, depending on the season, the total GWP per 1 kg of paddy rice is 1.34 kg CO_2_ eq (monsoon season) and 2.85 (dry season). This is because, during the dry season, producers have to grow rice by relying on underground irrigation. Likewise, even in Masuda’s (2019) study, where a rice crop in Japan is analyzed, the water used for planting is almost exclusively rainwater or water from canals; thus, the impact related to its extraction is not included. In general, it emerges how, for most of the studies, the main problem is precisely related to the use of fertilizers, in addition to the fact that natural water availability strongly influences the final impacts. There are also some Italian LCA studies that identify the main critical issues in rice production. In particular, it has been shown how water management of rice fields (Zoli et al. 2021) or how straw management (Fusi et al. 2014) affects CH_4_ emission. In addition, studies have been conducted on how different types of fertilizers (Fusi et al. 2017) or how organic farming techniques (Bacenetti et al. 2016) influence environmental impacts related to rice cultivation in northern Italy. To date, however, studies related to the environmental compatibility assessment of rice in Italy remain limited.

### Aim of the study

The need to study and assess the environmental impacts of rice farming in Italy arises because of the role and importance it has always played for the country. In fact, for about 500 years, that is, since rice began to be grown in the Po Valley, it holds a unique social and productive reality in the Italian and European agricultural landscape, a reality that is a source of employment for many people. In addition, the presence of rice and related canals and ditches ensures the maintenance of water for long periods (especially in summer), providing a natural refuge for many bird species destined to migrate elsewhere (Enterisi 2020). But Italy’s varietal heritage is also unique in the world, especially for the characteristics of wholesomeness and food safety guaranteed by the supply chain (Enterisi 2020), which make Italy a leading country in terms of production and varieties (Italy is the only producer in the world of *Arborio*, *Carnaroli* and *Vialone nano*). Therefore, because of the role played by rice farming in Italy, both for ecosystems and for the country’s economy and development, and as a result of limited national scientific production, the objective of this paper was to investigate its sustainability. The aim was to highlight the environmental weight of traditional Italian rice cultivation in comparison with new agronomic techniques (e.g., dry sowing), evaluating and highlighting the critical points of these systems. Moreover, the study was then compared with other international studies, to assess the greater or lesser sustainability of Italian agronomic techniques compared to other countries and thus how different production methods may influence the overall impacts. This also made it possible to suggest possible mitigation strategies, with particular attention to the irrigation phase.

Rice production in Vercelli (Piedmont), specifically a farm in *San Germano Vercellese* (VC), was chosen as a case study, and the assessment was conducted through LCA, using the SimaPro 9.2.0 software. The inventory data was analyzed with ReCiPe 2016 method, and the hierarchist (H) time perspective was chosen among the three proposed by the ReCiPe method because this is based on the most common policy principles concerning timeframe and is the most balanced one. Then, to analyze different cultivation practices, the possibility of combining the results of different LCA studies with multivariate analysis (MVA) was considered, to compare our study with studies previously conducted on rice cultivation in Italy.

## Materials and methods

### Goal and scope definition

**Fig. 1:**
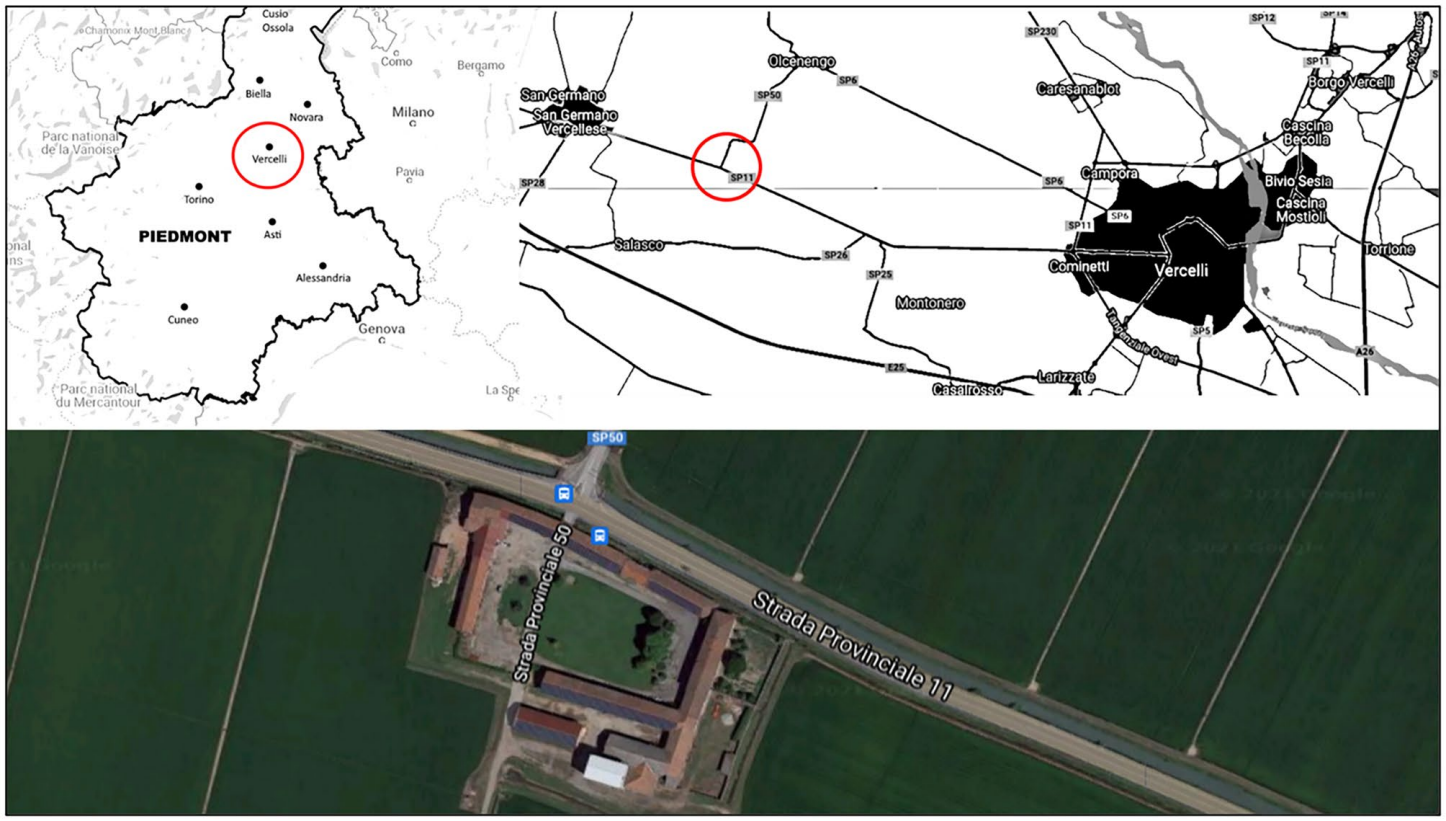
Geographical location of Vercelli rice production (studied production sites are highlighted)

The study’s goal is to assess the environmental performance of rice production in Northern Italy and thus identify the most critical hotspots and agricultural processes. In particular, as a case study (CS), a farm located in the province of Vercelli (45° 20′ 24.768′ N; 8° 17′ 17.17′ E) (Fig. 1) was chosen, whose province, together with Novara, represents the central rice district in Italy and Europe (Enterisi 2021). The chosen farm is family-run, with 2 workers, and produces about 7000 kg of rice per year, about 140 ha and 5000 h of work per year. The local climate is characterized by an average annual temperature of 12.5 °C, and rainfall occurs mainly in autumn and spring, with an annual average of around 745 mm. Therefore, in this area, rice is the leading annual crop and a fundamental source of income for farmers.

#### Functional unit (FU) and system boundaries

**Fig. 2:**
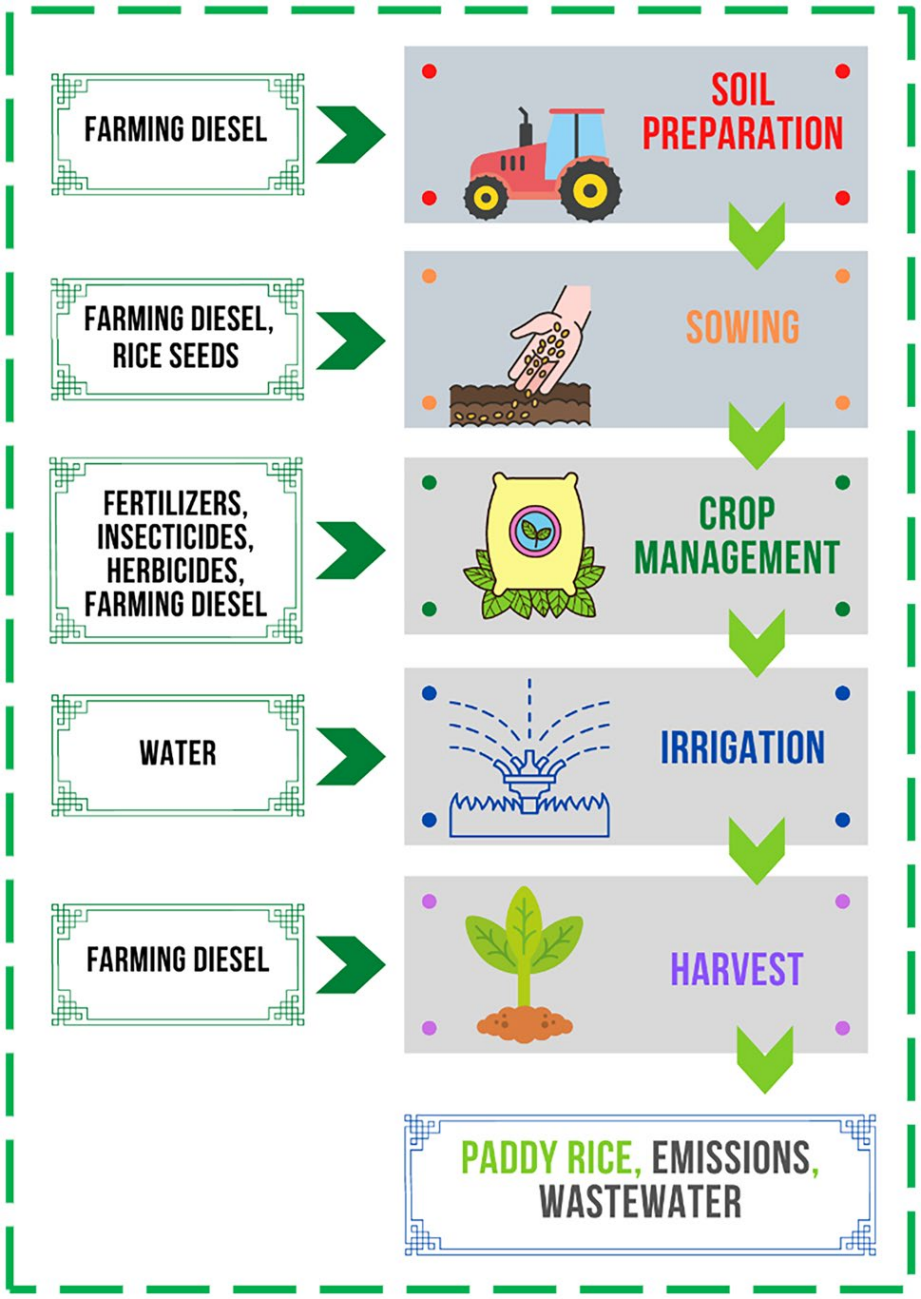
System boundaries: relevant inputs/outputs of Vercelli rice production

As FU, the cultivation of 1 hectare of land, which in the case study considered, provides about 7–8000 kg of rice, was chosen in this study. Rice production involves different inputs such as fertilizers, herbicides and insecticides, fuel, and water. Field operations require agricultural machinery for soil preparation, sowing, leveling, and application of fertilizers, herbicides, and insecticides, for which only fuel has been considered. The system boundary was chosen to be “from Cradle to Farmgate,” i.e., from the production of the seeds to the harvesting of the finished product (paddy rice), without considering seed transportation to the farm and storage, but considering the seed production process (Fig. 2). The choice of these system boundaries is mainly related to the fact that they are considered in most LCA studies in which the impacts of rice production are analyzed. For example, Escobar et al. (2022) who in their study considered all the processes involved in paddy rice production in Senegal (fertilizer and herbicide production, as well as production of diesel to operate machinery); Yu et al. (2022), Shen et al. (2021), and Chen et al. (2021) who assess the environmental impacts of some rice production in China, considering rice planting, irrigation, weeding, nutrition, and field operations, including the production processes of various inputs (fuels, pesticides, and various fertilizers) until the final product is harvested; or Rezaei et al. (2021), Houshyar et al. (2019), and Masuda (2019), who quantify the impacts of rice production in Iran (Rezaei et al. 2021 and Houshyar et al. 2019) and Japan (Masuda 2019), starting from the production of the inputs needed for production, to the harvesting of the finished product.

In general, mainly due to the availability of data and to dwell on the stages of the production process, there is a widespread tendency in LCA studies on rice production to take from cradle to farmgate as reference boundaries. However, what changes is that sometimes machinery production processes are also considered (Harun et al. 2021; Zoli et al. 2021) as well as transportation to the farm (Xu et al. 2022). In our study, the production process of the machinery was not considered, both because of data unavailability and because of its reduced environmental contribution due to its long useful life and depreciation (also in agreement with Xu et al. 2022 and Yu et al. 2022), as well as, again because of data unavailability, the other post-harvest production stages were also not considered. Therefore, for better alignment with other studies, and to focus on the impacts related to agricultural processes of rice production, the choice of boundaries fell on the from cradle to farmgate. The paddy rice production process consists of 8 phases (plowing, harrowing, leveling, sowing, fertilization, plant protection, irrigation and harvesting), which were grouped into 5 macro-phases: (1) soil preparation, (2) sowing, (3) crop management, (4) irrigation, and (5) harvesting.

### Life-Cycle Inventory (LCI)

The inventory data (Table 1) are mainly primary data representing a single average crop cycle covering the year 2020–2021. Specifically, primary data related to rice cultivation in our case study were collected through the administration of a questionnaire (Supplementary 1–2) obtaining information about the sequence of field operations as well as inputs consumed (such as pesticides, herbicides, fertilizers and water). The remaining data, such as agricultural diesel fuel for each stage, were collected from secondary data (ENAMA 2021).

**Table 1:** LCI of VRP

**Input**				
**Phases**	**Input**	**Typology**	**Unit/ha**	**Source**
**Soil preparation**				
Plowing	Farming diesel	Fuel	55.43 l	Ecoinvent 3
Harrowing			18.47 l	
Leveling			11.08 l	
**Sowing**				
Farming diesel		Fuel	9.24 l	Ecoinvent 3
Rice seeds		Production input	200 kg	Ecoinvent 3
**Crop management**				
Fertilization	Farming diesel	Fuel	17.15 l	Ecoinvent 3
	Organic NPK 10–5-15 mix	Fertilizer	400 kg	Ecoinvent 3
	UREA 46%		160 kg	Agribalyse 3
	Potassium Chloride (KCL)		160 kg	World Food LCA Database
Weeding	Farming diesel	Fuel	12.01 l	Ecoinvent 3
	Lambda-cyhalothrin	Insecticide	0.5 l	World Food LCA Database
	Cycloxydim pure (HRAC-A)	Herbicide	2.5 l	World Food LCA Database
	Imazamox (× 2) (HRAC-B)		2 l	
	Profoxydim (HRAC-A)		0.5 l	
	Florpyrauxifen-benzyl (HRAC-O)		1 l	
**Irrigation**				
Water		Production input	39,000 m^3^	Ecoinvent 3
**Harvest**				
Farming diesel		Fuel	38.79 l	Ecoinvent 3
**Output**				
Rice			7000 kg	
**Emissions to air**				
Carbon dioxide			7435.9 kg CO_2_ eq	
Methane			683 kg CO_2_ eq	
Dinitrogen monoxide			159 kg CO_2_ eq	
Sulfur hexafluoride			43.7 kg CO_2_ eq	
**Emissions to water (freshwater)**				
Phosphate			0.755 kg P eq	
Phosphorus			0.0533 kg P eq	
**Emissions to water (marine)**				
Ammonium, ion			0.0403 kg N eq	
Nitrate			0.56 kg N eq	
Nitrite			0.0001 kg N eq	
Nitrogen			0.0236 kg N eq	

Several tractors, ranging from 140 to 150 hp, are used in the various production phases and plows, harrows, and levels. Fertilizers, insecticides, herbicides, and diesel are used during the crop management phase. Among the fertilizers, 400 kg of mixed organic NPK (10–5-15), 160 kg of UREA 46, and 160 kg of potassium chloride; among the insecticides, 0.5 l of lambda-cyhalothrin; and among the herbicides, 2.5 l of cycloxydim pure, 2 l of imazamox for two applications, 0.5 l of profoxydim, and 1 l of florpyrauxifen-benzyl were used. As agricultural diesel, in the calculation, it was converted from liters into kg, using diesel density (0.820 g/cm^3^) as the conversion factor. In this study, the water consumption per hectare of one crop cycle of rice was considered (39,000 m^3^/ha) since the paddy field is flooded at a constant flow of 3 l/s/ha from mid-April to mid-August, and the excess water is fed into the adjoining paddy field. The amount of irrigation water (IW) was calculated using the formula described in the study by Mungkung et al. (2019) and expressed below in Eq. (1).

1 $$IW= \frac{\left(CWU-ER\right)+ WLP x 100}{IE}$$

where CWU is the crop water use in m^3^/ha, ER is the effective rainfall (m^3^/ha), WLP is the water loss percolation (m^3^/ha), and IE is the irrigation efficiency (m^3^/ha) of the irrigation system adopted. As mentioned in Sect. 2.1.1, machinery and plant production are excluded, both because of the unavailability of data and because the long useful life of plants plays a minor role as a contributor to environmental impacts. Data were then modeled on the databases in Simapro 9.2, especially, diesel, seed, NPK, and water from Ecoinvent 3 (Wernet et al. 2016), urea from Agribalyse 3 (Colomb et al. 2015), and potassium chloride (KCL), lambda-cyhalothrin (insecticide), and the four herbicides from World Food LCA Database (Nemecek et al. 2019). The Ecoinvent 3.4 database contains LCI data for energy production, transportation, chemical production, and fruits and vegetables. While Agribalyse 3 and World Food LCA Database are comprehensive LCI databases that have data for agricultural and agri-food products. Where possible, process data have been adapted to Italian conditions, since there are few data sets for Italian production processes. Therefore, most of the data used in this study are based on average process data from the international databases mentioned above (since they are the only ones available in the LCA software package used in this study) and adapted to be as consistent as possible with the objective and scope of the study.

### Life Cycle Impact Assessment (LCIA)

To have an assessment spectrum of the environmental performance of the analyzed company, the ReCiPe 2016 Midpoint (H) has been used. This methodology was chosen and preferred over other calculation methods such as ILCD 2011, CML 2001, or TRACI because having the availability of eighteen impact categories (compared to 16 of ILCD 2011 Midpoint, 15 of IMPACT 2002 + , 11 of CML-IA Baseline, and 9 of TRACI) can provide more comprehensive, articulate, and specific results on the environmental impacts of rice production than other methodologies with fewer impact categories. Therefore, Recipe 2016 Midpoint could give a broader picture with a greater degree of detail on the environmental impacts of production. Confirming this, it also appears to be the most commonly used methodology in LCA studies of rice production. In fact, from a Scopus literature review, in the last 5 years, about eight authors (Xu et al. 2022; Escobar et al. 2022; Rezaei et al. 2021; Shew et al. 2019; Habibi et al. 2019; Harun et al. 2021; Yodkhum et al. 2018; Jimmy et al. 2017) have used this methodology. Therefore, as a matter of completeness and greater detail of environmental impacts, and to make the results of our study comparable with other literature studies, we chose to use the ReCiPe 2016 Midpoint (H) methodology. Emissions from the irrigation phase and from the nitrogen fertilizers were calculated in accordance with the Kyoto Protocol (IPCC 2006), which considers seven different types of greenhouse gasses (carbon dioxide, methane, nitrogen dioxide; hydrocarbons; hydrofluorocarbons, sulfur hexafluoride, and perfluorocarbons), which are expressed as CO_2_ equivalent emissions. The emission was calculated as in Eq. (2), in accordance with Forster and Artaxo (2007).

2 $$\mathrm {GHGs}=\sum {G.G.}_{I}\times {k}_{i}$$

where *G.G*.i is the amount of greenhouse gas produced, and *k*_*i*_ is the CO_2_ equivalent coefficient for that gas.

### Uncertainty analysis

To assess how variability in the inventory data affected the results, ISO recommends conducting an uncertainty analysis to support the rigor of the results. Currently, among the most widely used software for LCAs, including SimaPro, Gabi, and Open LCA, the Monte Carlo (MC) method is the only one implemented for this type of analysis, and although it can also be conducted through other methodologies (Heijungs 2020), in our case study, we chose the MC. It is a sampling-based method in which calculations are repeated many times in order to estimate the probability distribution of the outcome and then extrapolate features such as the mean and standard deviation (Heijungs 2020). For our case study, the execution of the MC analysis took place in several steps:

1. First, a probability density function was assigned for each input data, and to choose the best option, the statistical distributions were adjusted using the “goodness of fit” method. This allowed the data to be fit to normal or log-normal distributions.
2. Next, random sampling of the input data and choice of test number and confidence interval was performed
3. Then, probability distribution functions were generated
4. Finally, the results were interpreted

The Monte Carlo simulation was performed using the function implemented in the SimaPro 9.2.0 software, which automatically assigns distribution to inventory data, preferring log-normal distribution. Normally, the number of tests chosen in Monte Carlo analysis is arbitrary, ranging from 1000 to 10,000. As a matter of time and practical feasibility, we chose an average value of 5000 runs in our study. The distributions obtained gave results in terms of expected values, with a 95% confidence interval for each indicator in the average impact categories. The Monte Carlo method, in accordance with Zhai et al. (2021), is explainable by Eqs. (3–6):

3 $$\eta =\eta ({x}_{1}, {x}_{2},{\dots x}_{M})$$

4 $${\eta }_{i}=\eta ({x}_{1i}, {x}_{2i},{\dots x}_{Mi})$$

5 $$\overline{\eta }=\frac{1}{N}\sum_{i=1}^{N}\eta i$$

6 $${\sigma }^{2}= \frac{1}{N-1}\sum_{i=1}^{N}({{\eta }_{i}- \overline{\eta })}^{2}$$

where:

1. $\left({x}_{1i}, {x}_{2i},{\dots x}_{Mi}\right)$ represent the *i*th random samplings of the input data ${(x}_{1}, {x}_{2},{\dots x}_{M})$
2. η is an arbitrary output value, a function of the input data ${(x}_{1}, {x}_{2},{\dots x}_{M})$
3. ${\eta }_{i}$ =  the value of the output parameter decided by the *i*th random sampling
4. $\overline{\eta }$ is the average output of parameter η after N random samplings
5. σ^2^ is the standard deviation of the output parameter η after *N* random samplings.

### Sensitivity analysis

Sensitivity analysis is performed to assess what effects on the results occur when an input parameter is changed. A “local” approach was applied in this study. Local sensitivity analysis determines the effect of a “small” change in one input parameter at a time (Groen et al. 2017). The sensitivity analysis was conducted to evaluate how the change in the amount of water being supplied to the system results in changes in the impact categories at the midpoint level. The amount of water is one of the parameters considered most influential on rice production and therefore this affects the calculated impacts. Three different water management models in rice fields were considered for this analysis:

- Water-sowing continuous flooding (WSF), a technique in which paddy fields are inundated without anticipating dry periods;
- Dry-sowing-delayed flooding (DSF), this procedure involves dry-sowing and flooding the paddy field at the time of germination of the 3rd–4th rice leaf;
- Dry-sowing-intermittent irrigation (DSI): this type of rice irrigation involves alternating periods when the paddy is flooded and dry.

The data applied in the sensitivity analysis did not consider rainfall for the years of data collection (2018–2021), a parameter that greatly influences the amount of water required to flood the paddy field. Hydrological balance data, to assess the irrigation use of different water management, were obtained from a previous study on water management in paddy fields conducted in the same area as the present study (Abruzzese et al. 2014).

### Multivariate analysis

In this study, a multivariate statistical analysis was performed in order to reduce the complexity of the output data. Specifically, a principal component analysis (PCA) was conducted to reduce the number of variables describing a data set to a smaller number of latent variables, limiting data loss as much as possible. The application of this method allows us to rank the environmental performance of our case study. Statistical analysis was conducted using R software (version 3.0) between the output data of our case study and those of other case studies that have performed LCA analyses on rice cultivation in Italy, in particular, the studies by Fusi et al. (2014), Bacenetti et al. (2016), Fusi et al. (2017), and Zoli et al. (2021). These studies were taken into consideration as they analyzed rice cultivation in the same geographical area. Also, these studies differed in the type of rice cultivation considered. Bacenetti et al. (2016) have considered in their studies organic rice cultivation while the other studies consider traditional cultivation. However, Fusi et al. (2017) have considered traditional rice cultivation fertilized with urban sewage sludge, while Zoli et al. (2021) considered likewise the traditional cultivation too, but in this case, they have studied different water management characterized by a period of soil aeration.

## Results and discussions

### ReCiPe 2016 Midpoint (H)

**Fig. 3:**
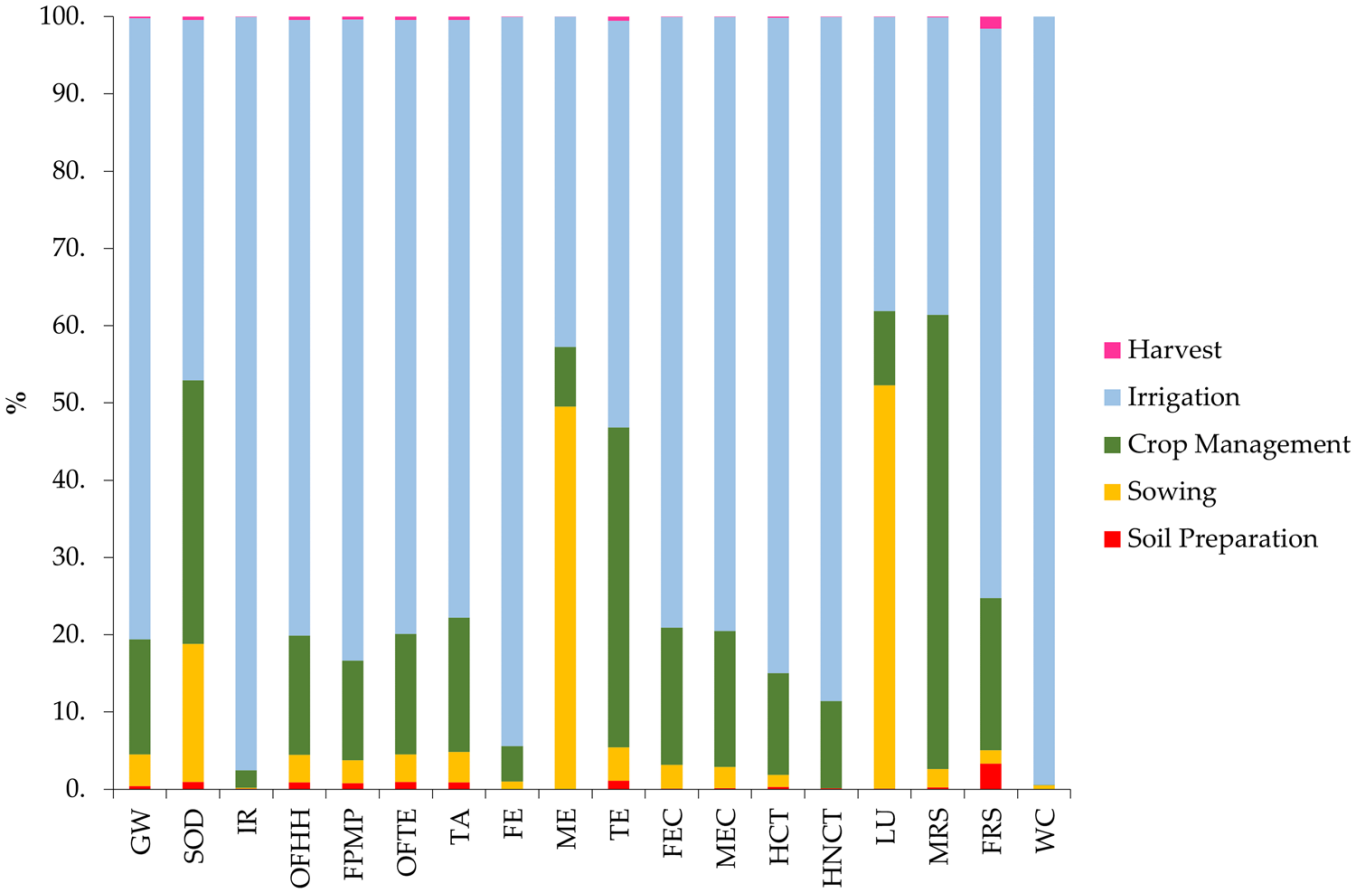
LCIA of VRP (characterized results)

The application of the LCA has allowed the study of rice production, highlighting the phases that have the most significant impact. The results of the assessment are shown in Table 2. Then the results have been characterized and expressed as a relative impact (Fig. 3).

**Table 2:** LCIA Results of VRP

**Impact categories**	**Unit**	**Soil preparation**	**Sowing**	**Crop management**	**Irrigation**	**Harvest**	**Total**
Global warming (GWP)	kg CO_2_ eq	3.35 × 10^1^	3.42 × 10^2^	1.24 × 10^3^	6.68 × 10^3^	1.53 × 10^1^	8.31 × 10^3^
Stratospheric ozone depletion (SOD)	kg CFC11 eq	6.31 × 10^−5^	1.19 × 10^−3^	2.28 × 10^−3^	3.11 × 10^−3^	2.88 × 10^−5^	6.67 × 10^−3^
Ionizing radiation (IR)	kBq Co-60 eq	2.46 × 10^0^	4.45 × 10^0^	7.63 × 10^1^	3.27 × 10^3^	1.12 × 10^0^	3.35 × 10^3^
Ozone formation, human health (OFHH)	kg NO_x_ eq	1.43 × 10^−1^	5.70 × 10^−1^	2.47 × 10^0^	1.28 × 10^1^	6.54 × 10^−2^	1.60 × 10^1^
Fine particulate matter formation (FPMF)	kg PM_2.5_ eq	9.66 × 10^−2^	3.53 × 10^−1^	1.55 × 10^0^	9.99 × 10^0^	4.41 × 10^−2^	1.20 × 10^1^
Ozone formation, terrestrial ecosystems (OFTE)	kg NO_x_ eq	1.52 × 10^−1^	5.78 × 10^−1^	2.53 × 10^0^	1.29 × 10^1^	6.94 × 10^−2^	1.62 × 10^1^
Terrestrial acidification (TA)	kg SO_2_ eq	2.86 × 10^−1^	1.25 × 10^0^	5.53 × 10^0^	2.46 × 10^1^	1.31 × 10^−1^	3.18 × 10^1^
Freshwater eutrophication (FE)	kg P eq	2.77 × 10^−3^	6.44 × 10^−2^	3.14 × 10^−1^	6.44 × 10^0^	1.26 × 10^−3^	6.82 × 10^0^
Marine eutrophication (ME)	kg N eq	2.92 × 10^−4^	5.29 × 10^−1^	8.23 × 10^−2^	4.57 × 10^−1^	1.33 × 10^−4^	1.07 × 10^0^
Terrestrial ecotoxicity (TE)	kg 1.4-DCB	1.13 × 10^2^	4.30 × 10^2^	4.14 × 10^3^	5.27 × 10^3^	5.15 × 10^1^	1.00 × 10^4^
Freshwater ecotoxicity (FEC)	kg 1.4-DCB	3.91 × 10^−1^	1.16 × 10^1^	6.74 × 10^1^	3.00 × 10^2^	1.78 × 10^−1^	3.80 × 10^2^
Marine ecotoxicity (MEC)	kg 1.4-DCB	6.92 × 10^−1^	1.39 × 10^1^	8.81 × 10^1^	3.98 × 10^2^	3.16 × 10^−1^	5.01 × 10^2^
Human carcinogenic toxicity (HCT)	kg 1.4-DCB	2.01 × 10^0^	1.04 × 10^1^	8.66 × 10^1^	5.57 × 10^2^	9.17 × 10^−1^	6.57 × 10^2^
Human non-carcinogenic toxicity (HNCT)	kg 1.4-DCB	1.22 × 10^1^	7.01 × 10^−1^	1.09 × 10^3^	8.54 × 10^3^	5.55 × 10^0^	9.65 × 10^3^
Land use (LU)	m^2^a crop eq	4.41 × 10^−1^	2.51 × 10^2^	4.61 × 10^1^	1.83 × 10^2^	2.01 × 10^−1^	4.81 × 10^2^
Mineral resource scarcity (MRS)	kg Cu eq	5.83 × 10^−2^	6.20 × 10^−1^	1.53 × 10^1^	1.00 × 10^1^	2.66 × 10^−2^	2.60 × 10^1^
Fossil resource scarcity (FRS)	kg oil eq	8.10 × 10^1^	4.13 × 10^−1^	4.77 × 10^2^	1.78 × 10^3^	3.70 × 10^1^	2.42 × 10^3^
Water consumption (WC)	m^3^	3.33 × 10^−2^	1.95 × 10^2^	2.88 × 10^1^	3.91 × 10^4^	1.52 × 10^−2^	3.93 × 10^4^

In this case, the results of the study, expressed in different units (kg CO_2_ eq, kg SO_2_, kg PM_2.5_, etc.) are multiplied by characterization factors and are expressed in terms of relative impact. That is, the sum of the emissions of the various phases is put as 100%, and the various individual results are calculated accordingly as relative impact. This type of visualization makes LCIA results more usable, especially when using calculation methods such as the ReCiPe 2016 Midpoint, which considers 18 impact categories with different units of measurement. This makes it possible to analyze the categories at the same scale and highlight specific trends. LCA shows that the environmental problem of rice production is mainly due to irrigation, which has a more significant impact than the other stages in 15 out of 18 categories (83%), including 12 out of 15 stages with impacts greater than 75%. For example, as in Global Warming Potential, irrigation, with 6.68 × 10^3^ kg CO_2_ eq out of a total of 8.31 × 10^3^ kg, accounts for 80% of the impacts. This result can be explained by the fact that the warm, water-saturated soil and nutrients exhaled by rice roots provide ideal conditions for methanogenesis in rice fields, and similar results have been reported by other Italian studies such as Fusi et al. (2014) and Bacenetti et al. (2016) and international studies such as Su et al. (2015) and He et al. (2018), which identified CH_4_ emissions as the main contributor to climate change.

But such impacts, in addition to methanogenesis, are also likely attributable to pumping for water extraction. In fact, irrigation processes are highly energy-intensive and have a high environmental impact due to the process of electricity production, which in the case of the Italian mix, despite coming for a 41.74% share from renewable sources, remains mainly composed of natural gas (CH_4_) (43.2%), coal (7.9%), nuclear (3.55%), oil (0.50%), and other sources (3.1%) (GSE 2020). Energy production, therefore, is based on the use of fossil fuels and is responsible for the direct emission of greenhouse gasses such as CO_2_ and N_2_O, which inevitably affect GWP, as well as particulate matter. These results are also confirmed in ozone formation–human health, ozone formation–terrestrial ecosystems and fine particulate matter formation, where irrigation impacts on average about 80% (1.28 × 10^1^ kg NO_X_ eq out on 1.60 × 10^1^ kg for OFHH, 1.29 × 10^1^ kg NO_X_ out on 1.62 × 10^1^ for OFTE and 9.99 × 10^0^ kg PM_2.5_ eq out on 1.20 × 10^1^ kg for FPMP), in line with the findings of Escobar et al. (2022), where energy consumption for irrigation turns out to be the primary source of OFHH and OFTE, causing 75% of these impacts. But these results are also confirmed in the fossil resource scarcity (FRS), where irrigation impacts about 74% (1.78 × 10^3^ kg oil eq out on 2.42 × 10^3^ kg) and in agreement with Xu et al. (2022), who show that energy consumption under conventional rice production in China had the largest impact (35–38%) on FRS. However, irrigation also has an impact on freshwater eutrophication (94%) and terrestrial acidification (77%). Regarding FE, the nitrogen in fertilizers as known, tends to dissipate, either into the water in the form of nitrates or into the atmosphere as nitrogen peroxide. Many nutrients and nitrates, therefore, probably influence the high freshwater eutrophication in groundwater and wastewater that ends up in rivers due to the irrigation phase. Mungkung et al. (2019) and Tayefeh et al. (2018) also identified that water emissions from fertilizer use are the main contributor to FE. Acidification values, on the other hand, could be caused by the emission of SO_2_, which comes from lignite and bituminous coal from mines that are used for the electricity generation mix, and consumed for water pumping (Dincer and Bicer 2018). But irrigation also affects the impacts related to toxicity, and this could be due to the fact that irrigation water in the flooding phase remains in prolonged contact with the soil in the rice fields, absorbing some of the toxic substances dissolved in it (e.g., fertilizers, pesticides, insecticides, etc.) that are released into the freshwater basins. Therefore, the impact of water management on the toxicity category is the result of toxic releases into the air, water, and soil (Van Hung et al. 2020). In detail, in the case of terrestrial ecotoxicity, the irrigation phase impacts 5.3 × 10^3^ kg 1.4-DCB Eq. (52% of the total) and crop management impacts 4.1 × 10^3^ kg 1.4-DCB Eq. (41%). In this case, the effects related to the latter phase are most likely linked to the specific use of urea, in agreement also with Xu et al. (2022), in whose study its use accounts for 44.5–90% of the impacts related to this category, but also to pesticide emissions to agricultural soil and the use of both sulfuric acid and steam during their conversion process (Borrion et al. 2012). On the other hand, regarding the irrigation phase, its effect on terrestrial ecotoxicity could be due to the disposal of drilling wastes following the extraction of natural gas for energy production, wastes that include toxic materials (Ahmadi 2018). Likewise, the same waste disposal, as well as the extraction of coal and other fossil resources, could be the cause of increased marine ecotoxicity, freshwater ecotoxicity, human carcinogenic toxicity, and human non-carcinogenic toxicity as a result of mainly the large mass of water that was moved (39,000 m^3^) and thus also the electricity that was used for its extraction, compared to the relatively minor use of pesticides (6.5 l total). Specifically, regarding marine ecotoxicity and freshwater ecotoxicity, the impact could come from groundwater contamination as a result of the extraction process of natural gas used for the electric mix (le Campion et al. 2020), which causes substances such as hydrocarbons, benzene, organic compounds, heavy metals, and other chemical compounds to leach into the groundwater, which then end up in agricultural waters (and thus stored in grain roots) or in drinking water, thus also affecting carcinogenic and non-carcinogenic toxicity. Finally, the only three categories in which irrigation is not the most impactful hotspot are marine eutrophication, land use, and mineral resource scarcity. As for marine eutrophication, the phase that affects this indicator the most is seeding (49%), with 5.29 × 10^−1^ kg N eq out of 1.07 × 10^0^ kg. This could be due to the fact that seed production was also considered in the seedling stage. This process requires inputs such as fertilizers, pesticides, and diesel fuel for machinery, which could directly affect marine eutrophication, especially since there is gaseous leakage and leaching into marine ecosystems during this stage, which leads to an increase in P and N in the water, resulting in excessive primary productivity (Yu et al. 2022). Just to confirm this, the seeding stage also affects land use by 52% (2.51 × 10^2^ m^2^a sq. yd. of the crop out of 4.81 × 10^2^ m^2^a sq. yd.), as a result of the high volume of soil that subtracts from seed production. In the literature, there are no studies that have considered the different agricultural stages of rice production, but only Abdul Rahman et al. (2019) considered the seed production stage, showing that it affects 1.90% of the impacts of the entire rice production process. As for mineral resource scarcity, the stage that has the most significant impact is crop management (59%) with 1.53 × 10^1^ kg Cu eq out of 2.60 × 10^1^ kg, and this is most likely due to the fertilizer and pesticide production process, which induces the depletion of mineral resources and critical raw materials used in the chemical industry. These results are in line with those of Yu et al. (2022), who show that the use of fertilizer instead of digestate could induce an increase in abiotic resources of + 56–65%. In general, the LCA shows that irrigation combined with fertilizers and pesticides (and the consequent release of active ingredients) is by far the activity that induces a massive environmental load. This is because long and continuous immersions, combined with the presence of organic fertilizers, generate CH_4_ emissions to the atmosphere and large amounts of indirect climate-altering gasses (CO_2_, NO_X_, PM_2.5_, etc.) and waste due to the production of energy used to pump the water itself. Several studies have been carried out in the literature analyzing the impacts of rice production in various parts of the world (Table 3). The studies found, covering the last 6 years, and considering only conventional rice productions, are for countries such as China, Malaysia, Thailand, Japan, Bangladesh, Iran, Brazil, Senegal, and Italy. However, it can be seen that different FUs are considered, from kg of paddy rice to hectare of land, and this could sometimes make comparison difficult. Therefore, wherever possible, a comparison was made so as to assess the sustainability of the studied production (VRP) compared to other countries. The reference value chosen, as a matter of relative importance, was the GWP.

**Table 3:** Comparison of literature studies (GWP)

**Ref.**	**Countries**	**GWP** **(kg** **CO**_**2**_ **eq)**	**FU**
Shew et al. (2019)	Bangladesh	1.30 × 10^0^–2.80 × 10^0^	1 kg
Jimmy et al. (2017)		3.15 × 10^0^	1 kg
Coltro et al. (2017)	Brazil	3.16 × 10^3^	1 ha
		4.57 × 10^2^	1 ton
Xu et al. (2022)	China	1.26 × 10^1^	1 ha
Shen et al. (2021)		1.67 × 10^1^	1 ha
Masuda (2019)	Japan	1.27 × 10^4^–1.49 × 10^4^	1 ha
Morandini et al. (2020)	Iran	4.27 × 10^2^	1 ton
Habibi et al. (2019)		2.98 × 10^2^	1 ton
Zoli et al. (2021)	Italy	1.30 × 10^3^	1 ton
Abdul Rahman et al. (2019)	Malaysia	1.39 × 10^3^	1 ton
Escobar et al. (2022)	Senegal	1.98 × 10^0^	1 kg
Mungkung et al. (2019)	Thailand	2.88 × 10^0^	1 kg
Yodkhum et al. (2018)		6.40 × 10^−1^	1 kg

It can be seen from Table 3 that GWP varies from country to country. This is mainly based on the growing season, production techniques, and inputs. For example, among the studies in which 1 ton of rice is taken as the FU, the results vary from Iran’s 2.98 × 10^2^ kg CO_2_ eq (Habibi et al. 2019) to Brazil’s 4.57 × 10^2^ kg CO_2_ eq (Coltro et al. 2017), Italy’s 1.30 × 10^3^ kg CO_2_ eq (Zoli et al. 2021), and Malaysia’s 1.39 × 10^3^ kg CO_2_ eq (Abdul Rahaman et al. 2019). From the results in Table 3, it can be seen that, for example, two countries with different cultivation techniques, such as Malaysia and Italy, have almost equal results. In fact, the Italian production system (Zoli et al. 2021) is irrigated and thus includes the large impacts related to electricity to run the pumps, while the Malaysian one relies almost exclusively on rainfall during the monsoon season (Harun et al. 2021). Similar results (but without the irrigation input in Malaysia) are therefore most likely attributable to the increased use of fertilizers and pesticides in Malaysia as a result of the need to increase yields to feed a population that relies almost solely on rice for its staple diet. Regarding, on the other hand, the choice of 1 ha as FU, it can be seen that in the case of China and Japan the results are almost similar, ranging from 1.26 × 10^1^ to 1.67 × 10^1^ kg CO_2_ eq for China (Xu et al. 2022; Shen et al. 2021) and 1.27 × 10^4^–1.49 × 10^4^ kg CO_2_ eq for Japan (Masuda 2019). Again, China and Japan are rich in water resources because they receive very high annual rainfall, and therefore, their water sources are sufficient to meet the water needs required for rice cultivation. Therefore, the effects related to water pumping cannot be considered, and the results diverge mainly because of the inputs considered within them. The GWP of our study, on the other hand, is about 8.31 × 10^3^ kg CO_2_ eq, thus 66–76% lower values. Thus, it can be seen that although irrigation-related impacts are also added in the Italian production (while in the Japanese and Chinese productions they are not), for the same FU, the GWP of the two Asian productions still remains higher than that of the Italian production. This is most likely due to the unbridled use of complex fertilizers and chemicals/synthetics used to maximize yield as much as possible. To give an idea, in recent years, precisely because of the massive use of chemical fertilizers, China has managed to feed more than 20% of the global population with less than 10% of the arable land (Sun et al. 2019), reaching levels of 446 kg of nutrients per ha (NBSC 2016), about 3.5 times the global average. Excessive fertilizer use, especially in China, according to van Wesenbeeck et al. (2021) is likely due to small land sizes, poor education, and low reliance on science but also to a lack of vision on the part of the Chinese government, which on years has conducted an inactive policy in reducing excessive fertilizer use. In Japan, on the other hand, the reason behind the indiscriminate use of fertilizers may be related to their low cost. Therefore, the results show that the rice production studied (VRP), with the same FU, has a lower environmental impact than those considered in China and Japan. The same cannot be said of rice production in Brazil (Coltro et al. 2017), where, although other post-harvest stages are also considered, it is shown that cultivation of 1 ha of land causes a GWP of 3.16 × 10^3^ kg CO_2_ eq. However, these results relate only to a specific technique used by the author (sprinkler irrigation), and given the lack of studies in the Brazilian territory, it is difficult to make a comparison; as well, for the same reason, it is also difficult to make comparisons with other studies of other countries, such as India, Indonesia, Vietnam, Myanmar, and Philippines, which are among the world’s ten largest producers (Faostat 2020). In general, then, our study shows that in the Italian case study, the main contributor to environmental impacts is mainly related to excessive water use (which causes direct impacts related to methanogenesis and indirect impacts related to the energy production process for its pumping). The reason for this is also rooted in Italy’s soil and climate conditions, which are certainly not conducive to a type of cultivation comparable to those in Asia, where annual rainfall ensures sufficient water supply, but which instead requires continuous artificial water administrations. However, just as in the Asian countries considered, great concern also remains above all about the management of chemical nitrogen fertilizers, field emissions, and their production. Regarding the latter aspect, there is a clarification to be made. In Italy, the containment of agricultural pollution falls under the so-called nitrates directive (Directive 91/676/EEC 1991), which provides for a mapping of the territory in relation to the risk of nitrate pollution, based on the identification of “vulnerable” areas, and the setting of maximum allowable loads of nitrogen per hectare, differentiated in relation to vulnerability (for livestock manure it is 340 kg/ha in non-vulnerable zones and 170 kg/ha for vulnerable zones). Northern Italian regions, including Piedmont, Lombardy, and Veneto, have a large part of their territories considered vulnerable zones and are already required to implement nitrate level abatement programs, which, at least in theory, should induce farmers to limit or at least regulate their use, especially given the EU’s declaration that it wants to achieve nitrate abatement targets in water by 2027.

### Uncertainty analysis

The LCIA results should be as reliable as possible, but sometimes, there may be uncertainties due to incomplete knowledge, inaccurate measurements, or no/poor data quality. Therefore, a Monte Carlo simulation was performed to identify the impact categories most affected by uncertainty in the input data. Typically, uncertainty is calculated for each impact category by dividing the standard deviation by the mean (McMurray et al. 2017), and according to the IPCC, uncertainty is significant if the result is > 0.3. Table 4 shows the main results of the uncertainty analysis, namely: mean and median, standard deviation (SD), coefficient of variation (CV) (ratio of standard deviation to the mean), standard error of the mean (SEM) (standard deviation of the sample distribution of the mean), and uncertainty value.

**Table 4:** Result of the uncertainty analysis using Monte Carlo simulation at the midpoint level

**Impact categories**	**Mean and median**	**SD**	**CV**	**SEM**	**Uncertainty**
Global warming	7670.00	0.2110	0.28%	0.0029900	0.00002751
Stratospheric ozone depletion	0.01	0.0000	1.03%	0.0000000	0.00010269
Ionizing radiation	3320.00	0.0003	0.00%	0.0000046	0.00000010
Ozone formation–human health	14.50	0.0002	0.13%	0.0000027	0.00001324
Fine particulate matter formation	11.30	0.0002	0.20%	0.0000032	0.00001973
Ozone formation–terrestrial ecosystems	14.70	0.0002	0.14%	0.0000029	0.00001388
Terrestrial acidification	29.20	0.0004	0.13%	0.0000053	0.00001291
Freshwater eutrophication	6.73	0.0000	0.01%	0.0000001	0.00000133
Marine eutrophication	1.10	0.0005	4.30%	0.0000067	0.00042818
Terrestrial ecotoxicity	7860.00	0.2010	0.26%	0.0028400	0.00002557
Freshwater ecotoxicity	333.00	0.0007	0.02%	0.0000093	0.00000197
Marine ecotoxicity	442.00	0.0008	0.02%	0.0000106	0.00000170
Human carcinogenic toxicity	590.00	0.0005	0.01%	0.0000075	0.00000089
Human non-carcinogenic toxicity	9340.00	0.1130	0.12%	0.0016000	0.00001210
Land use	455.00	0.1120	2.47%	0.0015900	0.00024615
Mineral resource scarcity	79.40	0.0043	0.54%	0.0000607	0.00005403
Fossil resource scarcity	2210.00	0.0046	0.02%	0.0000650	0.00000208
Water consumption	39,300.00	0.0123	0.00%	0.0001740	0.00000031

Table 4 shows how for all categories considered the uncertainty is low, being for all < 0.3. The results show that all impact categories have low variations in values and, therefore, there is good reliability of the data (De Marco et al. 2018). Therefore, the results of our study are reliable and the uncertainty related to the choice and variability of the data does not significantly affect the results for the impact categories analyzed.

### Sensitivity analysis

**Fig. 4:**
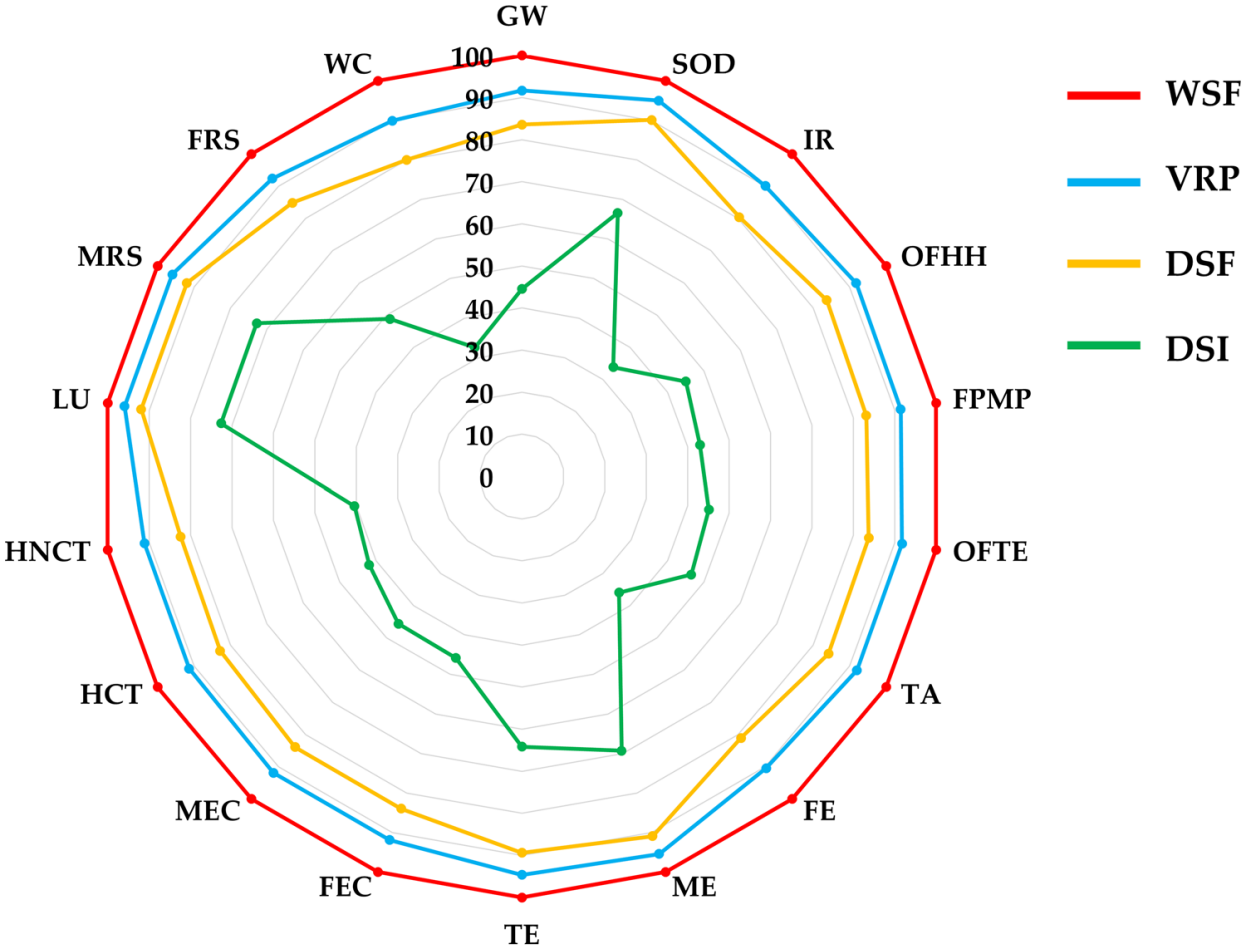
Sensitivity analysis at midpoint level for different water system management (GW, global warming; SOD, stratospheric ozone depletion; IR, ionizing radiation; OFHH, ozone formation–human health; FPMP, fine particulate matter formation; OFTE, ozone formation–terrestrial ecosystems; TA, terrestrial acidification; FE, freshwater eutrophication; ME, marine eutrophication; TE, terrestrial ecotoxicity; FEC, freshwater ecotoxicity; MEC, marine ecotoxicity; HCT, human carcinogenic toxicity; HNCT, human non-carcinogenic toxicity; LU, land use; MRS, mineral resource scarcity; FRS, fossil resource scarcity; WC, water consumption)

A sensitivity analysis was conducted to assess how changing certain input parameters would change the environmental performance of the VRP. Improvement options focused especially on water use because of the significant impacts identified in the LCIA. Sensitivity analysis is not regulated by ISO (Aziz and Hanafiah 2020), so alternative scenarios were created to demonstrate possible examples of improvement. Specifically, the analysis performed considers the change in the amount of water input to the paddy field under three different water management systems proposed by Abruzzese et al. (2014) and shown in Sect. 2.5. In our case study, water use in the paddy field follows the traditional method, which involves continuous flooding throughout the growth period of the rice plant, while in the alternative scenarios considered by us, three water management systems are proposed in which paddy fields are inundated without anticipating dry periods (WSF), planting is done dry while the paddy is flooded at the time of germination of the 3rd–4th rice leaf (DSF), and the paddy is flooded and dry with intermittent irrigation (DSI). The characterized results of the sensitivity analysis are shown in Fig. 4. It can be seen that the water management-based technique involving alternating periods of drought and field flooding (DSI) results in a cut in all 18 impact categories, with reductions ranging from 23–56% with an average of − 43%. The largest changes are observed for water consumption (− 57%), ionizing radiation (− 56%), and freshwater eutrophication (− 55%). Ionizing radiation is permanently present throughout the environment, and much of the average annual radiation dose received by people comes from natural environmental sources. However, it also comes from man-made sources such as nuclear power generation. In the Italian electricity mix, 3.55% of energy is generated by nuclear power plants (GSE 2020); therefore, a reduction in IR is most likely attributable to lower electricity use and thus plausible emission savings related to the nuclear share. Regarding FE on the other hand, a reduction in it could be due to the fact that lower water use could reduce the number of nitrates and nutrients that end up in freshwater and groundwater due to agricultural wastewater. But significant changes are also observed for human non-carcinogenic toxicity (− 52%), human carcinogenic toxicity (− 50%), and fine particulate matter formation (− 49%). In these three cases, with intermittent water management, the results were halved, and most likely this can be explained by the fact that less water use induces less use of electricity and thus consequent lower N_2_O emissions due to its production (explaining a − 49% reduction in FPMP) but also by reduced groundwater contamination caused by the extraction process of natural gas used to produce the electric mix, inducing less pollution of agricultural and drinking water, thus positively affecting carcinogenic (− 50%) and non-carcinogenic (− 52%) toxicity. For the same reasons, there is also a reduction in marine ecotoxicity and freshwater ecotoxicity (− 47%). On the other hand, regarding global warming, ozone formation–human health and ozone formation–terrestrial ecosystems, they too all show a reduction of − 47%, probably due to both direct effects such as lower methanogenesis in the field and lower greenhouse gasses (CO_2_ and N_2_O above all) caused by the electricity mix production process. In contrast to the DSI management system, the DSF system shows a reduction of about − 10% for all impact categories compared to this case study, while in the WSF system, the variation in impacts is greater than in the other water management systems (thus being less sustainable compared to VRP, DSF, and DSI), and this could be related to rice growing conditions. In fact, in this water management system, excluding a short period to allow for weed control, the paddy field is kept under constant flooding until pre-harvest drainage (Orasen et al. 2019). The sensitivity analysis thus shows how the use of the DSI management system could significantly reduce environmental impacts, in most categories by halving them, compared to the system used in this case study. Furthermore, the DSI system, although the system that uses the least amount of water, would appear to keep yields constant and in some cases even increase them compared to traditional irrigation techniques (Linquist et al. 2015). The other two systems (DSF and WSF) are not preferred over VRP, as they imply lower reductions in impacts than DSI (DSF) or even an increase compared to our case study (WSF). Thus, the sensitivity analysis shows how water management is a parameter that significantly influences the environmental impacts related to rice cultivation, both due to a reduction in direct impacts (less methanogenesis, less wastewater discharge) and indirect impacts (related to the production process of the electric mix). Good management of water resources in rice fields would result not only in the reduction of emissions related to this crop but also in a better adaptation of this type of cultivation to the sudden climate changes, which are characterized by increasing drought in this climatic zone (Zampieri et al. 2019).

### Multivariate statistical analysis

To better understand the results of the study and their relation to the different impact categories, principal component analysis (PCA) was applied. This analysis was conducted by comparing this case study with four case studies on rice production in which LCA was applied (Fusi et al. 2017, 2014; Zoli et al. 2021; Bacenetti et al. 2016). Table 5 shows the loadings of the principal component (PC) variables applied to the data set for all the case studies examined.

**Table 5:** Principal component loading for the impact category at midpoint level

**Impact categories**	**PC 1**	**PC 2**	**PC 3**
Global warming	−0.02485	0.15268	0.77781
Stratospheric ozone depletion	0.10799	−0.39692	0.006417
Ionizing radiation	0.27248	0.007848	0.35907
Ozone formation, human health	0.16735	0.35693	−0.02368
Fine particulate matter formation	0.13067	−0.38392	0.034671
Ozone formation, terrestrial ecosystems	0.16065	0.36245	−0.02393
Terrestrial acidification	0.098286	−0.40124	0.030931
Freshwater eutrophication	0.30861	−0.02005	0.036758
Marine eutrophication	0.30317	−0.04874	−0.18986
Terrestrial ecotoxicity	0.25781	0.15559	−0.00016
Freshwater ecotoxicity	0.30885	0.046974	0.007515
Marine ecotoxicity	0.30914	0.046253	0.007288
Human carcinogenic toxicity	0.31072	0.030664	0.093906
Human non-carcinogenic toxicity	0.31262	0.019958	0.048739
Land use	0.29861	−0.07348	−0.14109
Mineral resource scarcity	0.095739	0.19236	−0.43913
Fossil resource scarcity	−0.05742	0.41526	−0.03619
Water consumption	0.30404	−0.0664	0.001289
Variance %	56.51	31.05	7.48

**Fig. 5:**
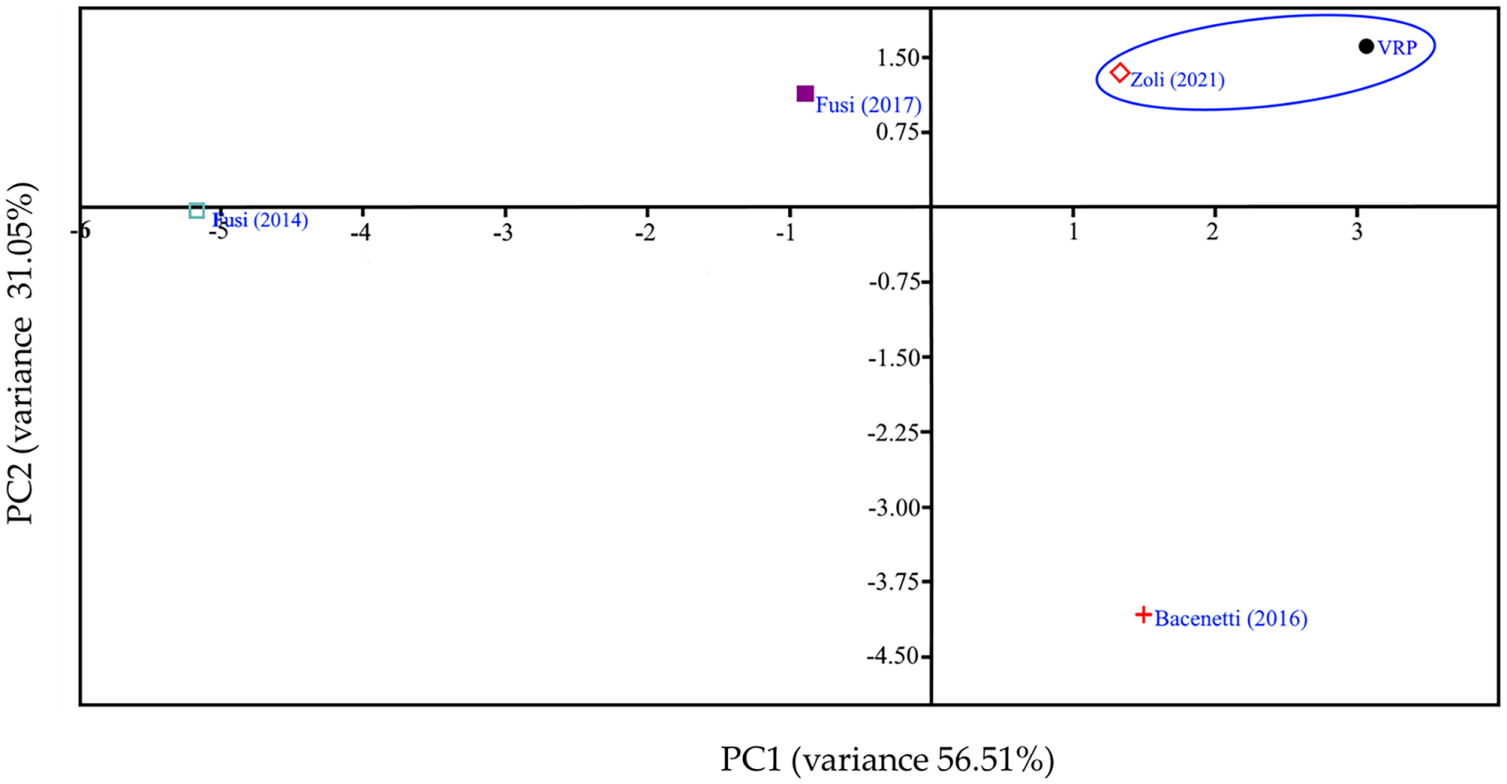
Score plot in the PC1-PC2 space

The PCA showed that the first three principal components accounted for 90% of the variance explained. PC1 (56.51% variance) strongly correlates positively and negatively with all impact categories. For example, the first principal component collects mainly the effects on FE, FEC, MEC, HCT, HNCT, and WC. PC2, which describes 31.05% of the variance, correlates positively with FRS and negatively with SOD, FPMP, and TA. In order to better explain the PCA analysis, the graph showing the scores concerning PC1 and PC2 has been reported (Fig. 5). From the scree plot, it is worth noting that this case study and the one conducted by Zoli et al. (2021) fall in the same position in the component space, indicating that these two agricultural processes present an equivalent environmental profile. On the other hand, the study of Bacenetti et al. (2016), conducted on the organic cultivation of rice, differs from the other studies conducted, which all deal with rice grown with conventional methods, positioning itself in the second quadrant (high PC1–low PC2). However, the limited number of statistical units allows only a statistical description of the data set to be carried out, while still providing a basic methodology for analyzing an output LCA data set.

## Conclusions

The objective of this study was to investigate the environmental impact of rice farming in Italy, and therefore, a farm in Vercelli was chosen as a case study. LCA was chosen for the analysis, using the ReCiPe 2016 Midpoint (H) calculation methodology, in order to have more comprehensive results. The results of the study show that the phase with the greatest environmental burden is irrigation, which compared to the other phases has a greater impact in 15 out of 18 categories, including 12 with impacts greater than + 75%. The LCA data has been analyzed through uncertainty analysis (Monte Carlo method), which showed that all impact categories have low values variations, thus good reliability. Generally, it emerges that the main problem can be traced right back to water management, especially since irrigation causes direct impacts, related to methanogenesis that occurs in rice fields, but also indirect impacts related mainly to the production of the energy mix needed to move the large masses of water needed for irrigation. All of this is in turn due to Italian soil and climate conditions, which necessitate an irrigated type of irrigation, as opposed to what happens in Asia, where annual rainfall and the dense network of canals and streams are sufficient to meet water needs. Subsequently, where possible, the results of our study were compared with other literature studies from other countries, and it emerged that the rice production considered induces less climate change than other conventional crops located in China and Japan, mainly due to less intensive use of fertilizers, the use of which in Italy is regulated by European Directive 91/676. Therefore, to understand what the best agronomic techniques might be to reduce the environmental impacts of irrigation, a sensitivity analysis was conducted, comparing different water management systems in the paddy field. The sensitivity analysis showed that the DSI water management system, characterized by intermittent flooding of the paddy field, has the least environmental impact, while the WSF system, with continuous flooding without dry periods, is the least sustainable. Therefore, the agronomic technique that would allow a reduction of the impacts (− 40% of the total impacts) is based on intermittent flooding of the rice fields compared to the high irrigation systems, including the one analyzed in the study. Furthermore, given the growing problems related to the reduction of rainfall in the Piedmont-Lombardy Po Valley area during the rice growing period, the intermittent irrigation system would allow the underground aquifers to be recharged, guaranteeing the preservation of the “water” resource, limiting and reducing the excessive losses of water that would occur with the continuous submersion of the rice, but also guaranteeing the possibility of supplying water to all agricultural crops in the area even in dry periods. Based on the results of our study, if sustainable agro-systems are to be developed to improve the environmental performance of rice farming, research will have to move in several directions, especially to optimize water resource management and fertilizer management, so as to decrease the share of greenhouse gasses, including CH_4_ due to methanogenesis, CO_2_ related to the production of electricity mixes, N_2_O related to fertilizer production, as well as other related indirect effects shown by the LCA results of our study. In particular, with regard to irrigation management, one possible area of improvement could be, for example, as also suggested by literature studies, to use alternative water management, introducing additional aeration periods and intermittent flooding of paddy fields, alternating wetting and drying, so as to reduce both methanogenesis and, where possible, the amount of water, thus inducing savings in terms of electricity as well. But simultaneously, straw and processing waste could be harvested so as to reduce the substrate from which CH_4_ could form and use it as a source of nutrients. At the same time, with a view to better fertilizer management, more organic soil conditioners could be used, including, for example, liquid digestate and compost, since the nutrients and organic matter in them make them particularly optimal for agricultural use, since their microbial activity increases carbon in the soil, improving its fertility. The reuse of straw (which is often burned), compost and liquid digestate, could fit into the circular economy (CE) perspective, as in this way, there could be an opportunity to provide more food but with fewer inputs. The CE, whose relevance in current research and policy is demonstrated by its central role in the European Green Deal and city action plans, calls for moving away from linear economy models, based on “take-produce-consume-discard,” and focuses on closing the circuits of raw materials, energy sources, and nutrients. In the agribusiness sector, given the large amount of organic waste (agro-waste) it produces, CE principles could be implemented quite effectively and efficiently. Indeed, in this context, some agro-wastes such as livestock manure, lignocellulosic agricultural biomass, but also liquid digestate, could be widely recommended and usable, as a full or partial replacement for synthetic fertilizers, as they can be bio-converted for food production. Likewise, compost derived from the organic waste of animal and plant origin could also provide all-natural nutrients and can thus function as an effective tool against erosion, sealing, loss of organic matter, loss of biodiversity, and contamination. In this regard, it should be pointed out that in Italy, there are 58 anaerobic digestion and composting plants producing compost and biogas, most of which are in the North (47), sometimes in the same regions where rice is produced, and this could help even more in the supply and exchange of agricultural waste, also with a view to industrial symbiosis. In addition, compost has a very low price, and is sometimes given away to farmers, who could therefore also benefit from financial savings. Therefore, by taking advantage of circular economy principles in rice farming, it might be possible to optimize and reduce the production of raw materials, replacing them with waste products, and consequently reducing pollutant emissions and where possible, making products more competitive. Finally, with regard to electricity production, which still takes place largely from fossil fuels, the energy crisis following the war in Ukraine should push countries even more to accelerate on the green transition. However, already in the aftermath of the COVID-19 pandemic, the transition to renewable energy had slowed down for the first time, and of the €14 trillion spent by G20 countries on economic stimulus measures, only 6% went to cutting emissions. Moreover, in the European Union, energy production from coal increased by + 18% in 2021, the first time in a decade, and in the next 20 years, Russia will most likely continue to export to Europe via the Nord Stream pipeline. At this rate, according to the latest IPCC report, we are heading toward a + 3.2 °C rise in temperatures by 2100, more than double the 1.5 °C that states have repeatedly pledged to achieve. Instead, research and investment will have to move in the opposite direction, through the creation of plants that provide high-density renewable energy, which cannot be obtained 100% from renewables, since the energy they provide is sometimes intermittent. Investment in renewables is, therefore, necessary precisely because high gas prices and inflated hydrocarbon prices make alternative sources economic in the long run and also because the need for energy security increases the urgency of addressing climate change.

## Limitations and future recommendations

In our study, the results were compared with those of other rice productions in some countries such as China, Japan, and Brazil. Therefore, at the same time, a literature analysis was conducted, from which some critical issues emerged.

First, different functional units and system boundaries are used among the various studies, with each author including or not including certain inputs (e.g., machine and plant production) and considering more or fewer production steps (distribution, packaging), with the results possibly being over- or underestimated at times. Therefore, making comparisons between literature studies is often not easy, and in our case, for comparison, we selected the studies that in terms of functional units, system boundaries and methodological framework were most similar to ours. But making comparisons is also not always possible because the limited scientific literature related to the application of LCA in rice farming covers a few countries, including China, Japan, and Bangladesh. In turn, within these very large countries, there are few studies related only to specific regions or production areas. Therefore, for these reasons, it was only possible to compare our results with a few other studies.

Then, it is also surprising how from some of the largest producers in the world (India, Vietnam, Myanmar, USA, etc.), there are few LCA studies investigating the impacts of their productions. Therefore, more studies will be needed from those countries with high production.

Finally, it is believed that for us to talk about sustainable rice production, it will also be necessary for future research to examine not only the environmental impacts of rice farming but also the social impacts through an increase in Social Life Cycle Assessment (S-LCA) studies, which rice farming research currently lacks. The study of social factors could prove to be a remarkable tool especially for local policies to empower women and the health and safety of workers.

## Supplementary Information

Below is the link to the electronic supplementary material.

Supplementary file1 (DOCX 92 KB)

Supplementary file2 (DOCX 23 KB)

## Data Availability

Data generated is publicly available and cited in accordance with the journal’s guidelines. All data used to support the results, as well as publicly archived data sets, analyzed or generated during the study were cited within the text. There are no ethical, legal, or privacy issues; there are no limitations in the declaration on data availability.
