# Supplementary material for: Environmental life cycle assessment of rice production in northern Italy: a case study from Vercelli
Source: Int J Life Cycle Assess. 2022 Oct 26:1–18. Online ahead of print. doi: 10.1007/s11367-022-02109-x (PMC9607803; doi:10.1007/s11367-022-02109-x)
Supplement: Supplementary file 1 — Supplementary file1 (DOCX 92 KB) [file 11367_2022_2109_MOESM1_ESM.docx]

**SUPPLEMENTARY MATERIALS. APPENDIX 1**

**
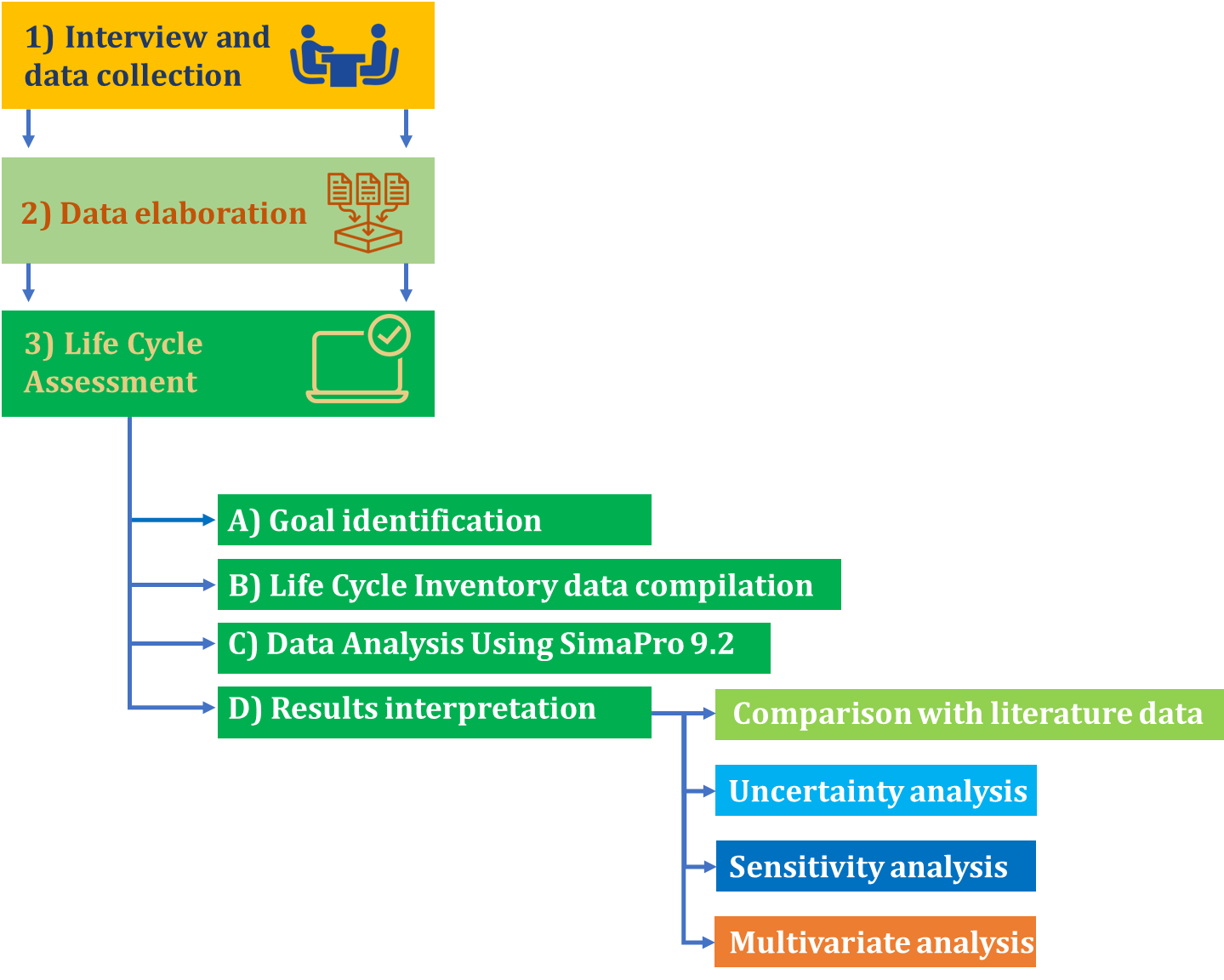
**

**Figure S1** – Flowchart for paper writing

1. **Interview and data collection:** During this phase, a form was prepared and administered to the owner of a Vercelli farm. The form administered can be seen in Supplementary 2 (Excel file)
2. **Data elaboration:** The data were then collected and processed (Supplementary 3), i.e., broken down by stages, as shown in the LCI
3. **Life Cycle Assessment:** The data were then modeled and entered into SimaPro 9.2 software. Next, the following were interpreted through Comparison with literature data (Scopus database), Uncertainty and Sensitivity analysis (SimaPro 9.2), Multivariate Analysis (MVA) (R software)
