# Supplementary material for: Environmental life cycle assessment of rice production in northern Italy: a case study from Vercelli
Source: Int J Life Cycle Assess. 2022 Oct 26:1–18. Online ahead of print. doi: 10.1007/s11367-022-02109-x (PMC9607803; doi:10.1007/s11367-022-02109-x)
Supplement: Supplementary file 2 — Supplementary file2 (DOCX 23 KB) [file 11367_2022_2109_MOESM2_ESM.docx]

**Table S1.** Data request information questionnaire

| FARM DATA | | | | | | | | |
| --- | --- | --- | --- | --- | --- | --- | --- | --- |
| Total hectares of the farm |  |  |  |  |  |  |  |  |
| Average paddy size (ha or m2) |  |  |  |  |  |  |  |  |
| number of workers |  |  |  |  |  |  |  |  |
| number of total working hours per year |  |  |  |  |  |  |  |  |
| RAW MATERIALS | | | | | | | | |
| Cultivated rice varieties (even 2/3 are enough) |  |  |  |  |  |  |  |  |
| Yield per 100 KG/ha |  |  |  |  |  |  |  |  |
| Amount of seeds planted per ha |  |  |  |  |  |  |  |  |
|  |  | Quantity | Fertilizer type | Time of distribution |  |  |  |  |
| Fertilizers | Nitrogen (N) |  |  |  |  |  |  |  |
|  | Phosphorus (P2O5) |  |  |  |  |  |  |  |
|  | Potassium (K2O) |  |  |  |  |  |  |  |
|  | Calcium (Ca) |  |  |  |  |  |  |  |
|  | Magnesium (MgO) |  |  |  |  |  |  |  |
|  | Silicon (SiO) |  |  |  |  |  |  |  |
| Other fertilizers |  |  |  |  |  |  |  |  |
|  |  |  |  |  |  |  |  |  |
|  |  |  |  |  |  |  |  |  |
|  |  |  |  |  |  |  |  |  |
|  | agropharmaceutical name | n°interventions | Quantity L per ha | Time of distribution |  |  |  |  |
| Herbicides |  |  |  |  |  |  |  |  |
|  |  |  |  |  |  |  |  |  |
|  |  |  |  |  |  |  |  |  |
| Fungicides |  |  |  |  |  |  |  |  |
|  |  |  |  |  |  |  |  |  |
|  |  |  |  |  |  |  |  |  |
| Indecticides |  |  |  |  |  |  |  |  |
|  |  |  |  |  |  |  |  |  |
|  |  |  |  |  |  |  |  |  |
| Average amount of water in the submersion phase (liters or cubic meters x ha) |  |  |  |  |  |  |  |  |
| Average amount of Water in the plant growth phase (liters or cubic meters x ha) |  |  |  |  |  |  |  |  |
| MACHINES | | | | | | | | |
|  |  | Machine used | Model (if possible) | Number of intervents | Machine working hours x ha (or only total working hours) | man's work hours x ha (or total working hours) | Power (horsepower) or (Kw) | Average diese (L) |
| Cultivation phases | Plowing |  |  |  |  |  |  |  |
|  | Harrowing |  |  |  |  |  |  |  |
|  | Leveling |  |  |  |  |  |  |  |
|  | Treatments (fertilization, weeding, etc.) |  |  |  |  |  |  |  |
|  | Submergence |  |  |  |  |  |  |  |
|  | Dry stage |  |  |  |  |  |  |  |
|  | Sowing |  |  |  |  |  |  |  |
|  | Harvest |  |  |  |  |  |  |  |
| Storage Phases | Silos |  |  |  |  |  |  |  |
